# Disease-Free Survival at 2 and 3 Years is a Significant Early Surrogate Marker Predicting the 5-Year Overall Survival in Patients Treated with Radical Cystectomy for Urothelial Carcinoma of the Bladder: External Evaluation and Validation in a Cohort of Korean Patients

**DOI:** 10.3389/fonc.2015.00246

**Published:** 2015-10-29

**Authors:** Hyung Suk Kim, Chang Wook Jeong, Cheol Kwak, Hyeon Hoe Kim, Ja Hyeon Ku

**Affiliations:** ^1^Department of Urology, Seoul National University College of Medicine, Seoul, South Korea

**Keywords:** urothelial carcinoma, radical cystectomy, surrogate, disease-specific survival, overall survival

## Abstract

**Purpose:**

We aimed to externally validate the association of 2- and 3-year disease-free survival (DFS) with 5-year overall survival (OS) in patients treated with radical cystectomy (RC) for urothelial carcinoma (UC) of the bladder.

**Materials and Methods:**

We reviewed the clinical data of 422 patients who underwent RC for UC of the bladder in our institution between 1991 and 2012. Survival curves were plotted with the Kaplan–Meier method. The Kappa statistic and Kendall tau-b test were used to assess the agreements between 2- and 3-year DFS and 5-year OS.

**Results:**

In the entire study population, 2- and 3-year DFS and 5-year OS rates were 76.4, 71.5, and 67.4%, respectively. All Kappa and Kendall’s tau-b test values for agreements between 2- and 3-year DFS and 5-year OS were more than 0.40, indicating moderate agreement for all patients and in each patient subgroup selected according to specific variables (all *p*-values <0.05). Kaplan–Meier analysis for DFS and Cox-proportional hazard models for landmark analysis at each time point indicated that most recurrences occurred within 3 years after surgery. The 5-year OS rates of patients who were recurrence-free at each time point gradually increased to more than 95% in an extended recurrence-free interval of 12–36 months.

**Conclusion:**

Our external validation results support the existing finding that 2- and 3-year DFS can be a valid early surrogate end point to predict 5-year OS after RC in patients with UC of the bladder.

## Introduction

Urothelial carcinoma (UC) of the bladder is a heterogeneous malignant disease with a variable clinical course and presentation (1). According to current guidelines, radical cystectomy (RC) with pelvic lymph node dissection (PLND) is the primary treatment option for muscle-invasive disease, which accounts for approximately 30% at initial diagnosis, and several high-risk non-muscle-invasive diseases, such as those refractory to bacillus Calmette–Guérin (BCG) therapy, histological variants of UC (i.e., micropapillary), and pT1 high-grade tumor with carcinoma *in situ* (CIS) (2–4). However, both the risk for undetectable occult micro-metastasis and tumor recurrence rates of 30–60% within 5 years after RC suggest that a unimodal treatment of RC may be insufficient for complete tumor control (1). Therefore, a multimodal approach, including neoadjuvant or adjuvant chemotherapy, should be considered for patients at high risk of postoperative recurrence (5).

Many studies address the impact of neoadjuvant or adjuvant chemotherapy on survival outcomes in UC of the bladder (5–7). While evidence from several meta-analyses exists for the use of neoadjuvant chemotherapy (cisplatin-based) in UC of the bladder (8, 9), data supporting adjuvant chemotherapy are suboptimal because of conflicting results (6, 7). Regardless of cancer treatment type, the goal of cancer treatment is to improve survival. Overall survival (OS) is widely used as the gold standard primary end point of clinical outcome in clinical trials for oncologic treatments, including surgery or systemic chemotherapy (10). The limitation of OS is the required long-term follow-up duration of >5 years. Therefore, investigators have sought to identify early surrogate markers that accurately predict OS following treatment (10–12). Previous reports suggest that disease-free survival (DFS) at 2 and 3 years (2- and 3-year DFS) represents an early surrogate end point that can replace or predict OS after surgical intervention or systemic chemotherapy in various malignancies, including UC (13–17). Because a majority of recurrences or metastases after RC generally occur within 3 years (18), it is probable that decrease in the disease-recurrence risk within 3 years following RC may improve the OS. According to this hypothesis, the use of the 2- and 3-year DFS as an early surrogate marker will facilitate the development of effective adjuvant chemotherapeutic regimens for RC.

In this study, we aimed to externally validate existing results (14, 15) that support the use of the 2- and 3-year DFS as a significant early surrogate end point for predicting 5-year OS following RC and PLND in patients with UC of the bladder.

## Materials and Methods

### Study Population

After obtaining institutional review board (IRB) approval from Seoul National University Hospital, we retrospectively reviewed the records of 486 patients in the bladder cancer database who received RC and PLND at our institution between 1991 and 2012. Patients who received postoperative radiation therapy (1 patient) and neoadjuvant chemotherapy (50 patients), and those with non-UC (13 patients) were excluded from this study. Ultimately, a total of 422 patients were eligible for our study.

### Data Acquisition and Definition of Variables

The indications for RC and PLND included muscle-invasive disease exceeding clinical stage T2 or those at high risk of non-muscle-invasive disease unresponsive to intravesical therapy (i.e., BCG instillation), such as high-grade Ta or T1 tumors and CIS. RC and PLND were performed as previously reported (19). Pathological specimens obtained from RC and PLND were examined by a professional genitourinary pathologist. Assessed pathological variables included pathological tumor (pT) stage and tumor grade, pathological nodal (pN) stage, perivesical margin, total number of removed lymph nodes and positive lymph nodes, and presence of CIS, lymphovascular invasion (LVI), and variant histology. Pathological staging and grade were determined according to the 7th (2010) edition of the Tumor-Node-Metastasis classification and the 2004 World Health Organization system, respectively. We re-investigated the pathological stage and grade of patients who underwent RC prior to current guideline systems. Perivesical margin was defined as the presence of residual tumor at the area of soft tissue on the final pathological slide of the RC specimen; therefore, urethral and/or ureteral margin status was not regarded as a margin. Other evaluated covariates were age, sex, body mass index (BMI), and adjuvant chemotherapy history.

### Follow-up

Postoperative follow-up was carried out according to institutional protocol. Follow-up occurred at least every 3–4 months for the first year, semiannually for the second year, and annually thereafter. Follow-up examinations included physical examination, blood test, chest roentgenography, kidney ultrasonography, and/or abdomen-pelvic computed tomography. A bone scan was performed only in cases with bone-related symptoms. DFS was defined as the interval between RC and disease recurrence or death from any causes. Post-RC recurrence at the ureter and/or urethra was not considered disease recurrence, but an additional primary event. The duration of OS was estimated from the date of RC to the date of last follow-up or death from any cause. Living patients were censored from relevant analyses. Death was identified by reviewing medical charts and/or from the annual census of the Korea National Statistical Office. Cause of death was determined by the responsible physicians and death certificates.

## Statistical Analysis

Continuous and categorical variables were, respectively, expressed as the median and interquartile range (IQR) and absolute numbers and relative percentages (%) in accordance with descriptive and frequency analyses. DFS and OS were estimated using the Kaplan–Meier method for the entire study cohort or subgroup stratified according to pT stage (pT2 or less versus pT3 or greater), and then compared using univariate log-rank tests between groups. We applied the Kappa statistic and Kendall’s tau-b test (20), which measure agreement between categorical variables, to elucidate the association of 2- and 3-year DFS with 5-year OS in all patients. In addition, correlation between the 2- and 3-year DFS and 5-year OS was evaluated at the individual level using Kappa statistics and the Cox-proportional hazard model. All statistical analyses were performed using the SPSS software version 21.0 (SPSS Inc., Chicago, IL, USA) and two-sided *p*-values <0.05 were considered statistically significant.

## Results

### Patient Characteristics and Outcomes after RC

Median age and BMI of all patients were 64.0 years (IQR: 57.5–70.0) and 23.3 kg/m^2^ (IQR: 21.2–25.1), respectively. The majority of patients were men (86.5%). There were 271 patients (64.2%) with pT2 or less and 151 patients (35.8%) with pT3 or greater disease. Adjuvant chemotherapy was administered in 102 patients (24.2%). Other clinicopathological characteristics are listed in Table 1.

**Table 1:** **Baseline characteristics of the study cohort**.

Variables	
No. of patients	422
Age (year), median(IQR)	64.0 (57.5–70.0)
≤65 years	224 (53.1%)
>65 years	198 (46.9%)
Gender, *n* (%)	
Male	365 (86.5%)
Female	57 (13.5%)
BMI (kg/m^2^), median (IQR)	23.3 (21.2–25.1)
<25	310 (73.5%)
≥25	112 (26.5%)
Tumor grade, *n* (%)	
Absence of cancer	50 (11.8%)
Low grade	20 (4.7%)
High grade	352 (83.4%)
Pathological T stage, *n* (%)	
pT0	50 (11.8%)
pTis	36 (8.5%)
pTa	21 (5.0%)
pT1	77 (18.2%)
pT2	87 (20.6%)
pT3	122 (28.9%)
PT4	29 (7.9%)
Pathological N stage, *n* (%)	
pN0	332 (78.7%)
pN1	37 (8.8%)
pN2	44 (10.4%)
pN3	9 (2.1%)
Presence of LVI, *n* (%)	
Absent	277 (65.6%)
Present	145 (34.4%)
Concomitant CIS at cystectomy, *n* (%)	
Absent	300 (71.1%)
Present	122 (28.9%)
Histological variant, *n* (%)	
No	389 (92.2%)
Yes	33 (7.8%)
Margin status, *n* (%)	
Negative	411 (97.4%)
Positive	11 (2.6%)
Adjuvant chemotherapy, *n* (%)	
No	320 (72.8%)
Yes	102 (24.2%)

*BMI, body mass index; LVI, lymphovascular invasion; CIS, carcinoma in situ*.

**Figure 1:**
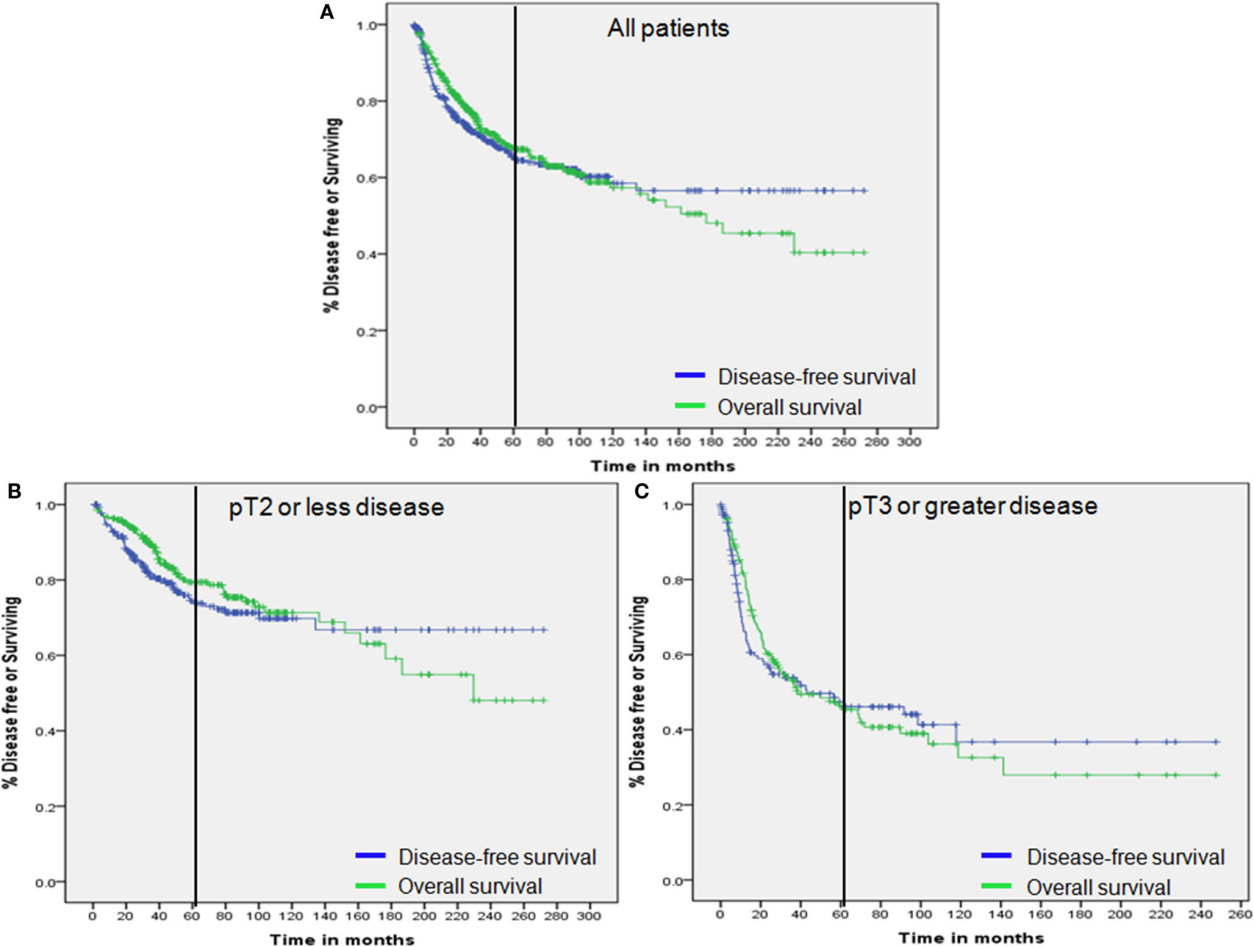
**Overall survival and disease-free survival**. **(A)** All patients. **(B)** Patients with pT2 or less disease. **(C)** Patients with pT3 or greater disease. Vertical bar represents 60 months.

The respective 2- and 3-year DFS and 5-year OS were as follows: 76.4, 71.5, and 67.4% in all patients; 85.9, 80.3, and 78.6% in patients with pT2 or less disease; and 56.4, 52.8, and 45.4% in patients with pT3 or greater disease (Figure 1). The recurrence rates within 2 and 3 years and the 5-year OS rates after RC were statistically significantly different between the two groups (all *p*–values <0.001).

In the entire study population, there were 133 cases of disease recurrence with a median follow-up time of 37.3 months (IQR: 13.8–81.8). Among all recurrences, 91 cases (68.4%) occurred within the first 2 years and 107 cases (80.4%) within the first 3 years following RC. Among patients with pT2 or less disease (*n* = 271), there were a total of 63 recurrences (23.2%) with a median follow-up time of 44 months (IQR: 24.4–87.0). In this group of recurrences, 35 cases (55.5%) occurred within the first 2 years and 47 cases (74.6%) within 3 years after RC. For patients with pT3 or greater disease (*n* = 151), there were a total of 70 recurrences (46.4%) with a median follow-up time of 14.9 months (IQR: 6.0–61.7). Among this group of recurrences, 56 cases (77.1%) occurred within the first 2 years and 60 cases (85.7%) occurred within 3 years after RC.

**Figure 2:**
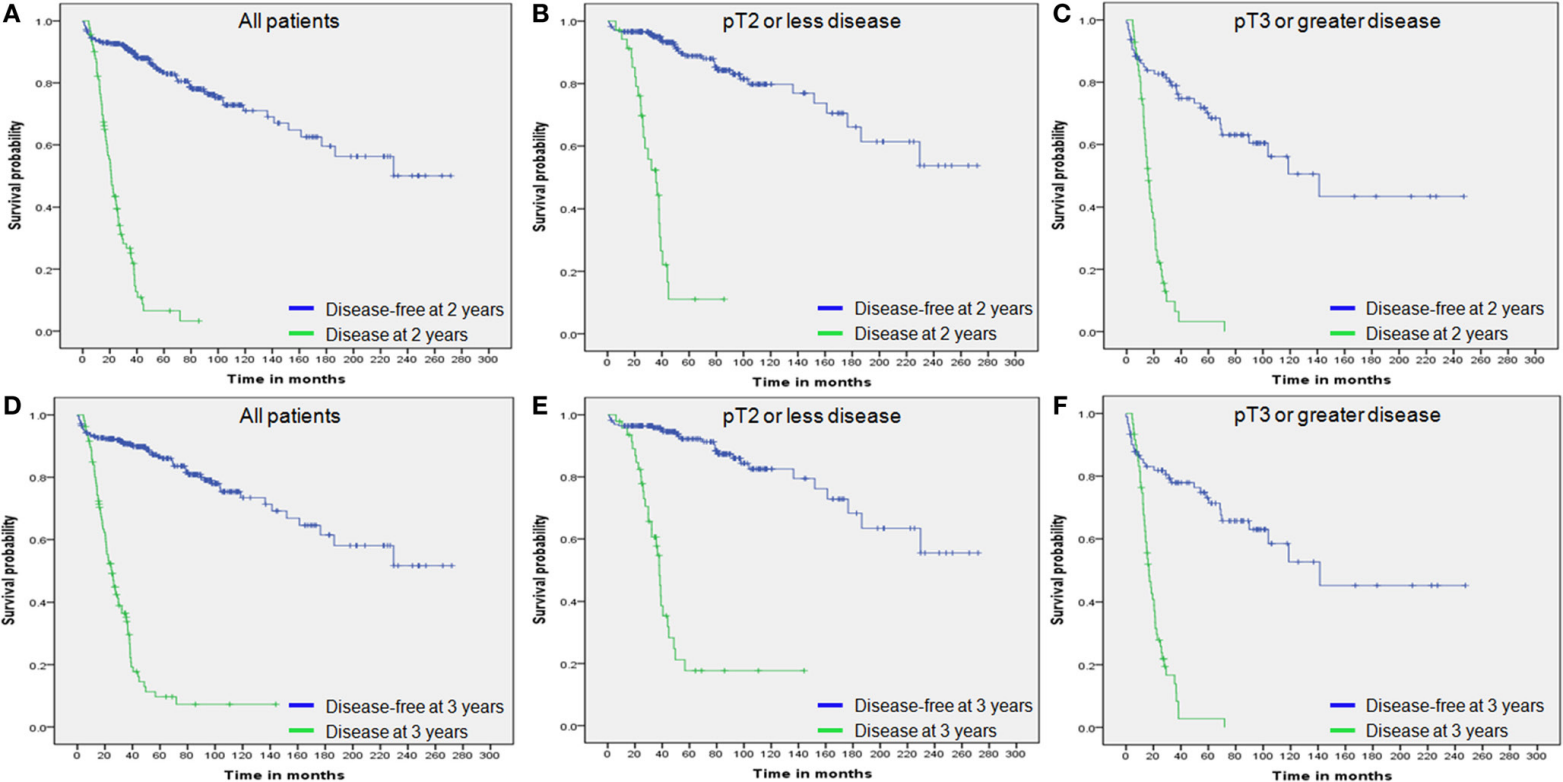
**Overall survival by disease status**. **(A)** Overall survival of all patients by 2-year disease status. **(B)** Overall survival of patients with pT2 or less disease by 2-year disease status. **(C)** Overall survival of patients with pT3 or greater disease by 2-year disease status. **(D)** Overall survival of all patients by 3-year disease status. **(E)** Overall survival of patients with pT2 or less disease by 3-year disease status. **(F)** Overall survival of patients with pT3 or greater disease by 3-year disease status.

Kaplan–Meier analysis with the log-rank test was used to analyze OS according to disease-recurrence status at 2 and 3 years following RC. For all patient groups including the entire study population, pT2 or less, and pT3 or greater, there were significant differences in OS rates according to time following RC (all *p*–values <0.001; Figure 2).

### Association of 2- and 3-year DFS and 5-year OS

The Kappa statistic and Kendall tau-b test were used to assess the agreements between 2- and 3-year DFS and 5-year OS. The statistical analysis determined values over 0.40 in each subgroup, in addition to the entire study population, implying at least moderate agreements for all relationships (all *p*-values <0.05; Table 2). In particular, substantial agreements (>0.60) between 2- and 3-year DFS and 5-year OS in the subgroup of patients that included men >65 years old and the presence of variant histology, absence of CIS, and use of adjuvant chemotherapy were observed.

**Table 2:** **The Kappa statistic and Kendall tau-b test for the dependence between disease-free survival and overall survival**.

	Kappa statistics (SE)		Kendall Tau-b (SE)	
	2-year DFS/5-year OS	3-year DFS/5-year OS	2-year DFS/5-year OS	3-year DFS/5-year OS
Total	0.59 (0.045)	0.62 (0.043)	0.60 (0.044)	0.63 (0.043)
Age ≤65 years	0.58 (0.069)	0.63 (0.063)	0.58 (0.068)	0.63 (0.063)
Age >65 years	0.60 (0.061)	0.61 (0.060)	0.61 (0.059)	0.61 (0.060)
Male	0.60 (0.047)	0.63 (0.046)	0.61 (0.046)	0.63 (0.045)
Female	0.51 (0.142)	0.53 (0.135)	0.51 (0.142)	0.54 (0.135)
BMI <25 kg/m^2^	0.57 (0.052)	0.61 (0.051)	0.59 (0.051)	0.61 (0.050)
BMI ≥25 kg/m^2^	0.66 (0.087)	0.68 (0.082)	0.66 (0.087)	0.68 (0.082)
pT2 or less	0.54 (0.072)	0.59 (0.066)	0.55 (0.072)	0.59 (0.066)
pT3 or more	0.56 (0.066)	0.59 (0.065)	0.57 (0.064)	0.60 (0.064)
LN negative	0.54 (0.060)	0.56 (0.058)	0.55 (0.058)	0.56 (0.058)
LN positive	0.55 (0.138)	0.73 (0.113)	0.56 (0.134)	0.73 (0.113)
Histological variant (−)	0.58 (0.049)	0.62 (0.046)	0.59 (0.048)	0.62 (0.046)
Histological variant (+)	0.64 (0.130)	0.64 (0.130)	0.66 (0.121)	0.66 (0.121)
LVI (−)	0.54 (0.069)	0.60 (0.063)	0.55 (0.067)	0.60 (0.063)
LVI (+)	0.58 (0.068)	0.58 (0.068)	0.59 (0.067)	0.58 (0.068)
CIS (−)	0.63 (0.050)	0.66 (0.048)	0.64 (0.048)	0.66 (0.047)
CIS (+)	0.45 (0.104)	0.50 (0.097)	0.45 (0.104)	0.50 (0.097)
ACH (−)	0.54 (0.057)	0.57 (0.055)	0.56 (0.055)	0.57 (0.054)
ACH (+)	0.68 (0.076)	0.73 (0.070)	0.68 (0.075)	0.73 (0.070)

*All p-values for Kappa statistics and Kendall Tau-b are <0.05*.

*LN, lymph node; LVI, lymphovascular invasion; CIS, carcinoma in situ; ACH, adjuvant chemotherapy*.

**Figure 3:**
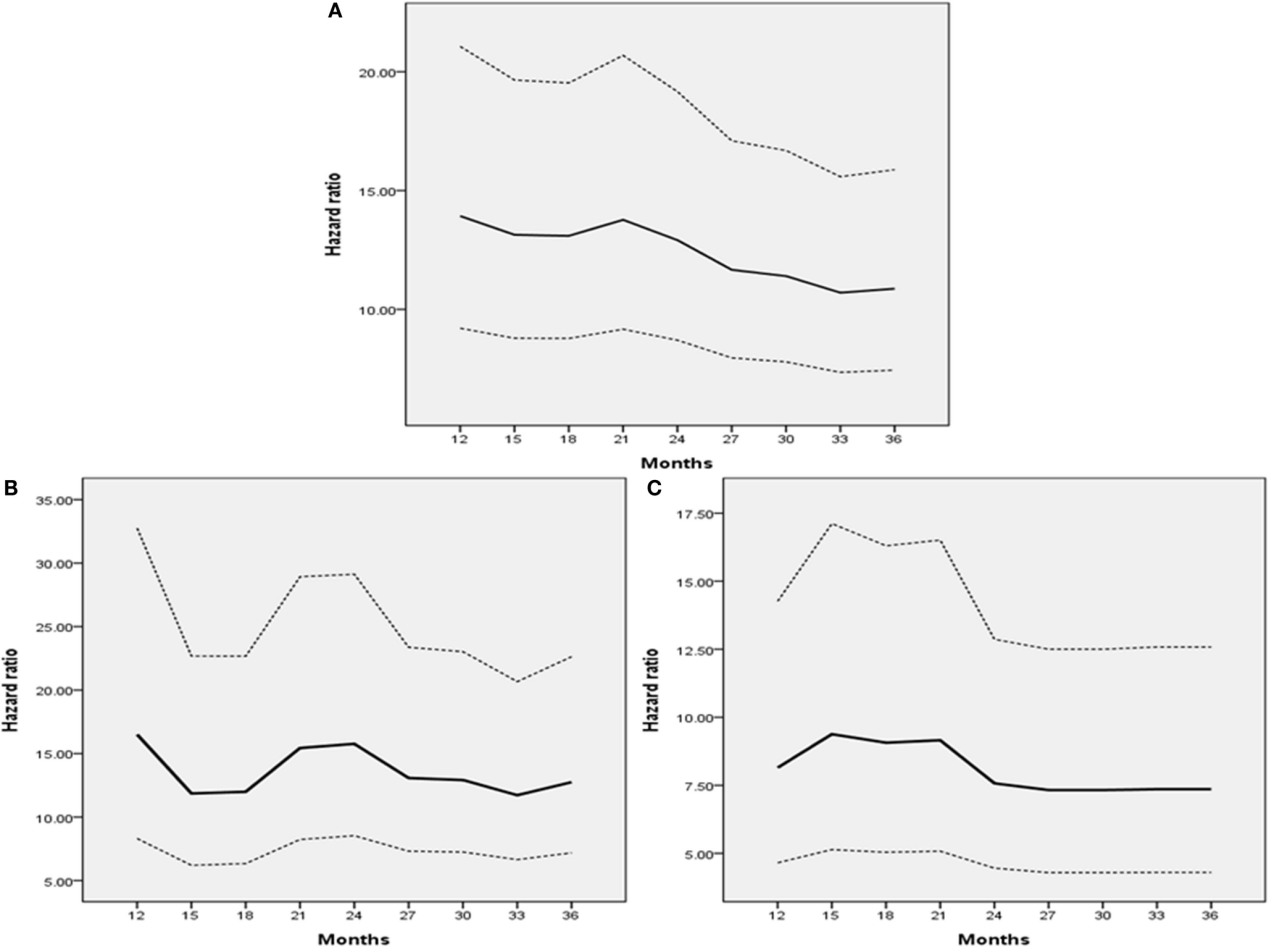
**Hazard ratio of landmark analysis at each time point**. **(A)** All patients. **(B)** Patients with pT2 or less disease. **(C)** Patients with pT3 or greater disease. Dashes indicate 95% confidence intervals.

Overall survival was compared in a landmark analysis that included a total of 64, 74, 76, 85, 91, 97, 99, 104, and 107 patients, who presented disease recurrence at postoperative 12, 15, 18, 21, 24, 27, 30, and 36 months, respectively. The hazard ratio (HR) between OS in patients with disease recurrence and those without recurrence at each time point is described in Figure 3. The HR gradually declined according to the extended disease-free interval and reached the lowest level at 36 months for the entire study population and the pT3 or greater disease group (Figures 3A–C). The lowest HR level was reached at 33 months in patients with pT2 or less disease (Figure 3B). These results and the 2- and 3-year DFS and 5-year OS curves (Figure 3 and Figure 1) indicated that most recurrences after RC could be accurately detected within an observational period of approximately 36 months.

**Figure 4:**
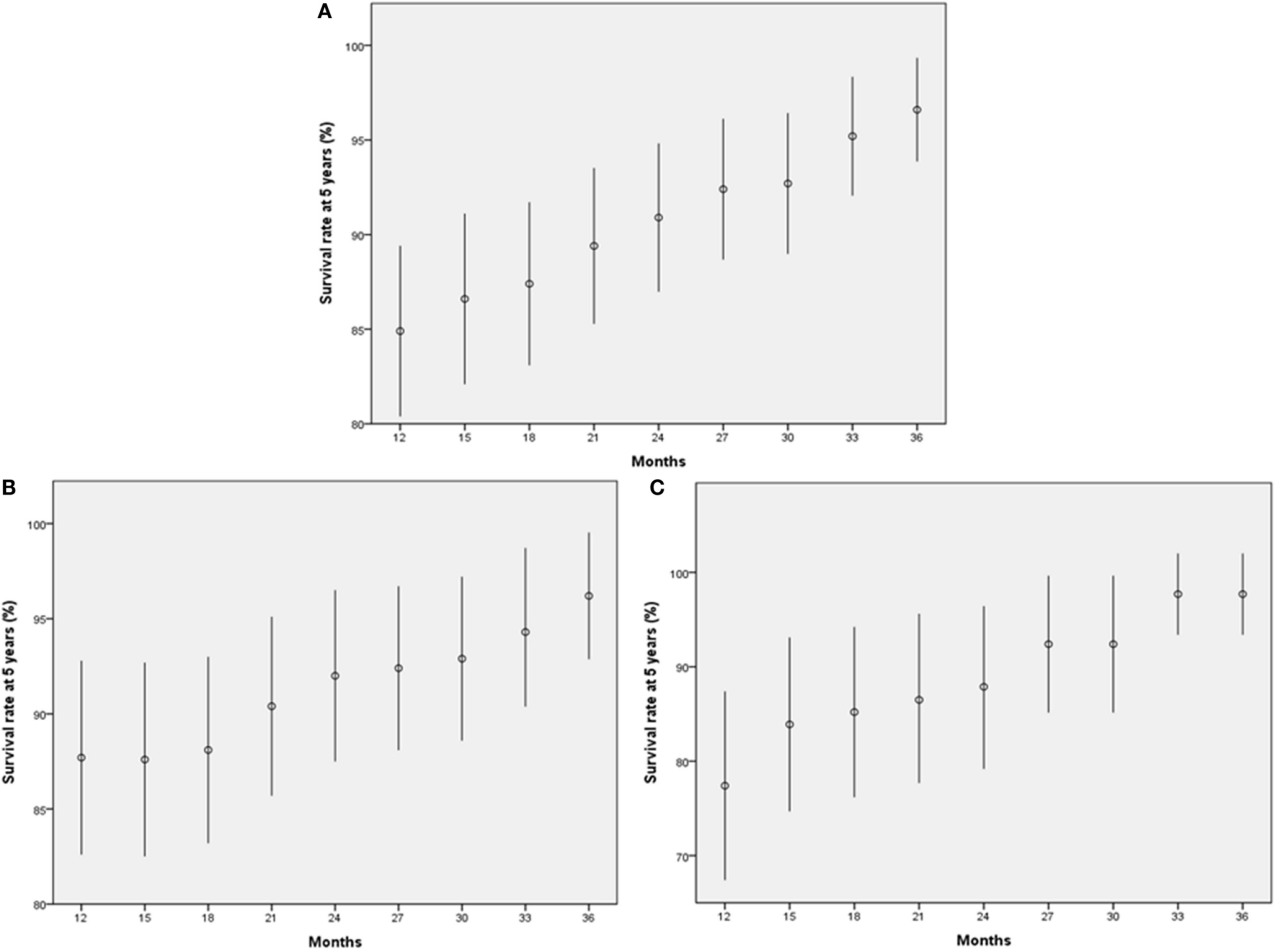
**Five-year survival rates of patients who achieved each outcome**. **(A)** All patients. **(B)** Patients with pT2 or less disease. **(C)** Patients with pT3 or greater disease. The bars indicate 95% confidence interval.

We also estimated the 5-year OS rates of patients who had no disease recurrence at each time point. In the entire study population, the 5-year OS rates of patients who were disease free at each time point (3-month interval from 12 to 36 months) gradually increased from 85% at 12 months to more than 95% at 36 months, depending on the extended recurrence-free interval (Figure 4A). Twenty-four months after RC, the 5-year OS rate exceeded 90%. When the entire population was dichotomized according to tumor stage (pT2 or less vs. pT3 or greater), the trend remained the same in each group (Figure 4B–C).

## Discussion

Overall survival is used in a variety of oncologic clinical trials as the primary end point to reflect the long-term efficacy and outcomes for surgical intervention, radiation therapy, and systematic drug treatment, including neoadjuvant and adjuvant chemotherapy, because of several associated advantages that include ease of measurement and interpretation, and its clinical significance (10). However, this end point may be a problem for prospective clinical trials assessing new anti-cancer agents because of the long waiting periods of ≥5 years to identify treatment-related efficacy. Therefore, early surrogates for the OS end point, which could be achieved more quickly, less invasively, and in a less costly manner than OS, are under investigation in a number of oncologic areas, including urologic malignancy (11–17, 21, 22).

Because most recurrences and disease progressions occur within the first 2 or 3 years after cancer treatment (15, 17, 18), 2- and 3-year DFS has been suggested as a valid predictor of 5-year OS. For example, a pooled analysis of phase III adjuvant colon cancer studies including 20,898 patients across 18 randomized trials revealed a high correlation between DFS and OS (13). This analysis led to the conclusion that DFS at the 2- and 3-year median follow-up should replace 5-year OS as the primary end point in future colon adjuvant trials. Akamatsu et al. demonstrated that progression-free survival at 2 years could be a reliable surrogate end point for 5-year OS in 159 patients treated with chemoradiotherapy for locally advanced non-small cell lung cancer (17). These associations between 2- and 3-year DFS and 5-year OS have also been reported in UC of the urinary tract. Sonpavde et al. evaluated the correlation of 2- and 3-year DFS with 2- and 3-year OS in a multicenter study, consisting of 2,724 patients treated with RC for muscle-invasive bladder cancer (14). They concluded that 2- and 3-year DFS is a potential intermediate surrogate for 5-year OS. This group also reported a similar significant association for 2,492 patients with upper tract UC who underwent radical nephroureterectomy (16). In each study discussed, the authors internally validated their findings using only the leave-one-out method. Nuhn et al. completed the first external validation of correlation between 2- and 3-year DFS and 5-year OS in patients with bladder cancer (15).

The results of our external validation study confirmed significant correlations between 2- and 3-year DFS and 5-year OS. When performing subgroup analysis according to a specific variable, the strength of this correlation was consistently maintained, especially in patients who received adjuvant chemotherapy. The association between 2- and 3-year DFS and 5-year OS in these patients showed substantial agreements with values >0.60. Similar to previous studies (14, 16, 17), most recurrences (more than 80%) occurred within the first 3 years after RC. Besides, in our study, the 5-year OS rates in patients without recurrence gradually increased at each time point in accordance with the extended disease-free interval. Patients with a disease-free interval >24 months showed a 5-year OS rate of >90%. According to our findings, DFS at 2 and 3 years may function as an alternative, short-term end point to 5-year OS when assessing the efficacy of novel chemotherapeutic agents as adjuvants in bladder cancer clinical trials. In other words, the goal of prospective novel anti-cancer drug development for advanced bladder cancer will focus on the improvement of DFS.

The present study is limited by several factors. First, the study’s retrospective, non-randomized design could not control for the presence of unidentified confounding factors. Our data were extracted from patients who underwent RC performed by multiple surgeons over a 20-year period. Guideline changes in surgical technique (i.e., PLND template) and pathological report content (i.e., variant histology) represent time-dependent variables that may have impacted our results. Furthermore, inter-physician variation based on surgery-related expertise, determination of adjuvant chemotherapy implementation, type of adjuvant chemotherapy regimen assigned, and postoperative follow-up strategies could affect the current study results. Although adjuvant chemotherapy regimen was not defined in the current study, the strong correlation observed between DFS and OS irrespective of adjuvant chemotherapy indicates that the association may not be regimen specific. Lastly, the study population was recruited from a single institution and included a relatively small sample size; therefore, the results derived from this study should be further validated externally using well-designed, randomized clinical trials.

## Conclusion

We externally validated the existing correlation between 2- and 3-year DFS and 5-year OS in patients who underwent RC for UC of the bladder. In particular, our validation suggests that 2- and 3-year DFS can be used as a potential early surrogate end point to predict or replace 5-year OS in future adjuvant bladder cancer clinical trials. Further external validation through well-designed prospective randomized trials will be required to confirm the role of DFS as the primary end point in bladder cancer.

## Author Contributions

Conception and design: JK, CJ, CK, HHK. Data acquisition: HSK, JK, CJ. Data analysis and interpretation: HSK, JK, CahCheol Kwak. Drafting the manuscript: HSK. Critical revision of the manuscript for scientific and factual content: Ja Hyeon Ku, CJ, CK, HHK. Statistical analysis: HSK, JK. Supervision: JK, CJ, CK, HHK.

## Ethical Approval of the Study

This study design and the use of patients’ information stored in the hospital database were approved by the Institutional Review Board (IRB) at the Seoul National University Hospital. The approval number is H-1412-073-632. We were given exemption from getting informed consents by the IRB because the present study is a retrospective study and personal identifiers were completely removed and the data were analyzed anonymously. Our study was conducted according to the ethical standards laid down in the 1964 Declaration of Helsinki and its later amendments.

## Conflict of Interest Statement

The authors declare that the research was conducted in the absence of any commercial or financial relationships that could be construed as a potential conflict of interest.
